# Prevalence of HIV among MSM in Europe: comparison of self-reported diagnoses from a large scale internet survey and existing national estimates

**DOI:** 10.1186/1471-2458-12-978

**Published:** 2012-11-14

**Authors:** Ulrich Marcus, Ford Hickson, Peter Weatherburn, Axel J Schmidt

**Affiliations:** 1 Department of Infectious Disease Epidemiology, Robert Koch Institute, P.O. Box 650261, Berlin, 13302, Germany; 2 Sigma Research, London School of Hygiene and Tropical Medicine, London, UK

**Keywords:** HIV prevalence, Men having sex with men (MSM), Seroprevalence studies, Internet survey

## Abstract

**Background:**

Country level comparisons of HIV prevalence among men having sex with men (MSM) is challenging for a variety of reasons, including differences in the definition and measurement of the denominator group, recruitment strategies and the HIV detection methods. To assess their comparability, self-reported data on HIV diagnoses in a 2010 pan-European MSM internet survey (EMIS) were compared with pre-existing estimates of HIV prevalence in MSM from a variety of European countries.

**Methods:**

The first pan-European survey of MSM recruited more than 180,000 men from 38 countries across Europe and included questions on the year and result of last HIV test. HIV prevalence as measured in EMIS was compared with national estimates of HIV prevalence based on studies using biological measurements or modelling approaches to explore the degree of agreement between different methods. Existing estimates were taken from Dublin Declaration Monitoring Reports or UNAIDS country fact sheets, and were verified by contacting the nominated contact points for HIV surveillance in EU/EEA countries.

**Results:**

The EMIS self-reported measurements of HIV prevalence were strongly correlated with existing estimates based on biological measurement and modelling studies using surveillance data (R^2^=0.70 resp. 0.72). In most countries HIV positive MSM appeared disproportionately likely to participate in EMIS, and prevalences as measured in EMIS are approximately twice the estimates based on existing estimates.

**Conclusions:**

Comparison of diagnosed HIV prevalence as measured in EMIS with pre-existing estimates based on biological measurements using varied sampling frames (e.g. Respondent Driven Sampling, Time and Location Sampling) demonstrates a high correlation and suggests similar selection biases from both types of studies. For comparison with modelled estimates the self-selection bias of the Internet survey with increased participation of men diagnosed with HIV has to be taken into account. For most countries self-reported EMIS prevalence is higher than measured prevalence, which is likely due to a combination of different time points of measurement, measurement errors for small sample sizes, different sampling methods, and an indicator-inherent overestimate of prevalence among the untested fraction of MSM.

## Background

International comparison of HIV prevalence among men who have sex with men (MSM) is challenging for a variety of reasons. Firstly, the denominator for such data, i.e. the absolute number of MSM in a given country, is unknown, and random samples for MSM cannot be drawn. Estimates on HIV prevalence among MSM thus are typically based on convenience samples [1] e.g.; for reviews see also [2-4]. Respective studies in post-industrialized countries have used samples of gay, bisexual, and other MSM recruited through virtual (such as websites or online social networks), or traditional venues for MSM, such as bars, sport-studios, etc. The different sampling frames that are used lead to methodological differences that directly impact the prevalence estimates and hence comparability. For venue-based sampling methods, number and type of venues may differ substantially within and between countries, and for snowball sampling methods like Respondent Driven Sampling, type and size of social and sexual networks may heavily depend on the social, political, and cultural acceptance of sexual minorities. On the other hand, self-reported data from anonymous Internet convenience samples are also subject to selection biases introduced through the sampling methods. Relevant to all measurement strategies is the size of the MSM population, which is defined in a variety of ways. The size of the HIV positive population depends on whether biological samples are tested or HIV diagnoses are self-reported. UNAIDS has suggested to use the proportion of those who have been diagnosed with HIV among those who have been tested for HIV as an indicator for HIV prevalence [5]. This assumes that those who have not been tested for HIV have the same HIV prevalence as those who have been tested, and - if applied for self-reported prevalence - it neglects those who have seroconverted since their last negative HIV test.

Participants in open access community-recruited Internet surveys are unlikely to be representative of the MSM population in any country. Surveys addressing sexual behaviour, sexually transmitted infections and HIV can be expected to have a self-selection bias towards men with a greater interest in sex and HIV, and of gay community attached MSM, particularly those diagnosed with HIV [6]. The high variability in participation rates in Internet surveys is probably related to external factors such as access to the Internet, popularity of Internet dating and contact sites often used to recruit survey participants for MSM in different countries, different relative sizes of the respective MSM populations, and other factors.

Participants of specific seroprevalence studies with formalized sampling frames like Time Location Sampling [e.g. [7] in gay venues or Respondent Driven Sampling [8] may be more representative of MSM attending such settings than seroprevalence studies without such formalized sampling frames, but still the claim to reach representative samples of the whole MSM population with these methods is unfounded, particularly because the absolute size and composition of the MSM population (which critically depends on the definition of MSM) remains unknown. This is underlined by the observation that repeated seroprevalence studies using such methods, e.g. in South-eastern European countries, sometimes result in declining seroprevalence estimates for HIV [9,10], suggesting changing self-selection biases for participation of men at risk for acquiring HIV over time.

Faced with the wide diversity of methods and approaches to measure HIV prevalence, an unresolved question is, whether and how data on HIV prevalence can be compared between different countries and studies using different methodologies.

To address this question, national data on HIV prevalence in the MSM population from a variety of European countries and using a variety of methodological approaches were compared with self-reported data on diagnosed HIV in survey participants of the first pan-European MSM Internet survey (EMIS) conducted in 2010.

## Methods

### EMIS derived data

A detailed description of the survey methods will be published elsewhere (a descriptive survey report including a description of methods will become available on the project website www.emis-project.eu in the first quarter 2013). Briefly, a network of five primary and 77 secondary partners working in MSM sexual health across academia, public health and community organizations in 38 European countries developed a collaborative English language survey. The survey was translated into 24 other languages, and prepared for administration on the Internet in a language of the users’ choice. It was promoted through gay online social media including PlanetRomeo, Manhunt, and Gaydar, and through gay community organizations. The survey was accessible online from June through August 2010. EMIS was approved by the Research Ethics Committee of the University of Portsmouth, United Kingdom (REC application number 08/09:21).

Survey participants were asked whether they had ever been tested for HIV, the result of their last test, and the year of their first positive result or most recent negative result. From these questions an indicator of HIV prevalence was constructed as recommended by WHO/UNAIDS for UNGASS (indicator number 23) and Global AIDS Response Progress reporting (indicator 1.14 [5,11])^a^:

HIV prevalence was defined as the proportion of respondents diagnosed HIV positive among those ever tested for HIV.

### Other HIV prevalence studies or estimates

We searched for HIV prevalence estimates and respective studies in the “Dublin Declaration Monitoring Report” published by ECDC and WHO/Europe [12], publications of other UN organisations [13], and on the UNAIDS country fact sheets [14] based on the UNGASS monitoring round in 2009.

(1) Prevalence estimates were derived from specific prevalence studies (directly measured prevalence in studies using venue-based, snowball or respondent driven sampling,

(2) self-reports in community based surveys (internet, bar/club or gay press recruited),

(3) calculated or modelled from the estimated total number of MSM living with HIV (based on surveillance data) and the estimated total size of the MSM population (from national statistics and general population surveys) using more or less sophisticated approaches [15,16].

Most prevalence estimates could be verified and partly updated by consulting the nominated contact points^b^ for HIV surveillance in the respective countries. Contact points from 32 countries responded, except Austria, Belorussia, Bulgaria, Croatia, Malta, and Moldova (for a list of the countries see Table 1).

**Table 1:** **National HIV prevalence data for MSM in European countries and corresponding self**-**reported prevalence data from EMIS**

			**Surveillance system**	**EMIS**	
		**source of HIV prevalence estimate; study methodology**	**HIV prevalence estimate; surveillance system %**	**Diagnosed HIV+ among ever tested (%)**	**Survey-Surveillance Discrepancy factor (SSD)**
excluded, no surveillance system prevalence estimates	Austria		n.a	7.2	
	Cyprus		n.a.	1.9	
	Ireland		n.a.	9.5	
	Malta		n.a.	2.5	
	Turkey		n.a.	3.0	
excluded, similar methodology	Belgium	2a	5.6	10.5	
	Switzerland	2a (2007)	8.1	11.5	
	France	2a (2009)	12.0	12.7	
	Sweden	2a (2008)	4.0	6.4	
specific prevalence studies	Bosnia	1b/c (<2008)	0.7	0.0	
	Bulgaria	2	3.3	2.5	0.7
	Belarus	2	2.7	3.0	1.1
	Czech	2b (2008/9)	2.6	6.7	2.6
	Estonia	2c (2007)	1.7	2.8	1.7
	Spain	2b (2008/9)	17.0	14.9	0.9
	Croatia	2	3.3	4.8	1.5
	Hungary	2	2.7	5.6	2.1
	Italy	1b (2008/9)	11.8	10.7	0.9
	Lithuania	2b	2.7	4.8	1.8
	Latvia	2b (2008)	4.0	7.8	1.9
	Moldowa	1b/c (2007)	4.8	4.3	0.9
	Macedonia	2b/c (2006)	2.8	7.7	2.7
	Poland	2b (2004)	4.7	8.3	1.8
	Portugal	3a (snowball)	11.0	10.9	1.0
	Romania	2b (2008/9)	4.6	5.5	1.2
	Serbia	3b/c (2008/10)	3.6	5.4	1.5
	Russia	2c	8.3	8.6	1.0
	Slovenia	2b (2008/9)	5.1	8.3	1.6
	Slovakia	2b (2008/9)	6.1	3.1	0.5
	Ukraine	3b/c (2011)	6.4	8.2	1.3
modelling/ health care system data	Germany	3e (2010)	4.9	11.6	2.4
	Denmark	1d (2009)	4.9	12.0	2.4
	Finland	3d (2009)	2.0	5.1	2.6
	Greece	1d (EPP)	6.5	12.9	2.0
	Luxemburg	3d (2010)	6.0	13.8	2.3
	Netherlands	1e (MPES 2007)	6.0	19.9	3.3
	Norway	3d (2008)	3.3	5.2	1.6
	United Kingdom	1/2e (MPES 2007)	5.3	14.6	2.8

1=UNGASS 2008; 2=UNGASS 2010; 3=personal communication.

a=self-reported; b=TLS, venue based; c=RDS; d=health care system; e=population-based modelling.

n.a.= not available.

### Comparison of prevalence study and survey derived prevalence estimates

Because of unknown self-selection biases in both prevalence studies and internet based surveys, no adjustments were made and prevalence study based estimates were compared directly with the EMIS prevalence estimate. If different prevalence estimates were available from seroprevalence studies for the same country (e.g. from different, not too distinct time points or different areas) a median value was calculated. For the six city SIALON study, which used time location sampling in larger cities of six Mediterranean and Central European countries [7], comparable regions were selected for the EMIS prevalence estimates.

For all countries a survey-surveillance discrepancy (SSD) factor was calculated by dividing the two prevalence rates derived from the EMIS survey and the prevalence studies: SSD=prev_EMIS_/prev_study_.

### Comparison of modelling and survey-derived prevalence estimates

For countries whose prevalence estimates were based on modelling or calculations involving surveillance data, the respective estimate of the relative or absolute size of the MSM population was used in the following formula to determine a survey-surveillance discrepancy (SSD) factor to get a crude measure for the self-selection bias in the Internet survey data:

(1) $$\begin{array}{cc} SSD=\frac{\frac{HIV_{\mathrm{EMIS}}}{N_{\mathrm{EMIS}}}}{\frac{HIV_{\mathrm{pop}}}{N_{\mathrm{pop}}}}= & SSD=\frac{HIV_{\mathrm{EMIS}}}{HIV_{\mathrm{pop}}}\cdot \frac{N_{\mathrm{pop}}}{N_{\mathrm{EMIS}}} \end{array}$$

In this formula HIV_EMIS_ is the number of survey participants diagnosed with HIV, HIV_pop_ is the estimated number of HIV-infected MSM in the population, N_pop_ is the estimated total size of the MSM population (for the countries with prevalence estimates based on modelled/surveillance data – Germany, Denmark, Finland, Luxemburg, Netherlands, Norway, UK – the relative size of the MSM population was assumed to be 3%, based on respective data from general population surveys from Germany and the UK), and N_EMIS_ is the respective national sample size in EMIS.

For each country the SSD represents the ratio of survey members diagnosed with HIV to the total sample size, divided by the ratio of the estimated number of MSM infected with HIV in the total population to the estimated total MSM population.

A value of 1 represents a country where survey data and surveillance data match. Values below 1 are countries where fewer men in the survey were diagnosed with HIV than would be expected from the surveillance data-based model (i.e. men with diagnosed HIV are under-represented in the survey, or over-represented in the surveillance data), and values above 1 are the reverse (i.e. men with diagnosed HIV are overrepresented in the survey, or underrepresented in the surveillance data).

## Results

A detailed description of the demographic characteristics of the participants is available online in the EMIS Final Report (accessible in early 2013 on http://www.emis-project.eu).

**Figure 1:**
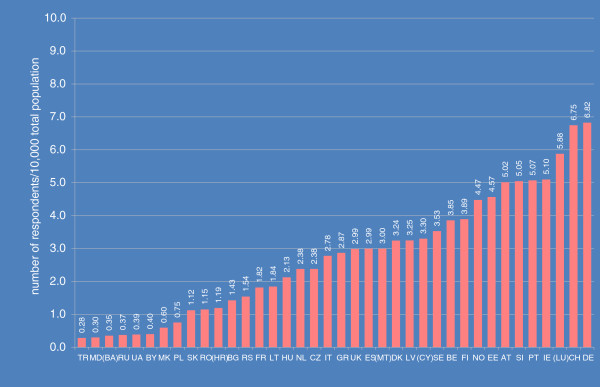
Participation rates in EMIS across the 38 countries with samples >100.

The proportion of the total population who participated in EMIS (*participation rate*) varied widely, from 3 to almost 70 per 100,000 (see Figure 1). As far as *response rates* per recruiter website could be determined – 109,951 out of a total of 174,209 respondents were recruited via personalized invitations from two supranational gay websites – differences in response rates were much smaller, between 4% and 14% of those invited to participate.

The proportion of EMIS participants who had ever been tested for HIV varied between 43% in Lithuania and 84% in France, the proportion of men with diagnosed HIV among those ever tested between 0% in Bosnia-Herzegovina and 19.7% in the Netherlands (see Table 1 and Additional file 1: Table S1).

Existing HIV prevalence estimates for MSM populations could be identified for 33 of the 38 EMIS countries (see Table 1). No prevalence estimates were identified for Austria, Cyprus, Ireland, Malta, and Turkey.

For four countries – Belgium, France, Sweden, and Switzerland – we only identified published estimates based on earlier national Internet surveys, methodologically very similar to EMIS. Because of the use of the same sampling methodology these four countries were excluded from further comparison.

**Figure 2:**
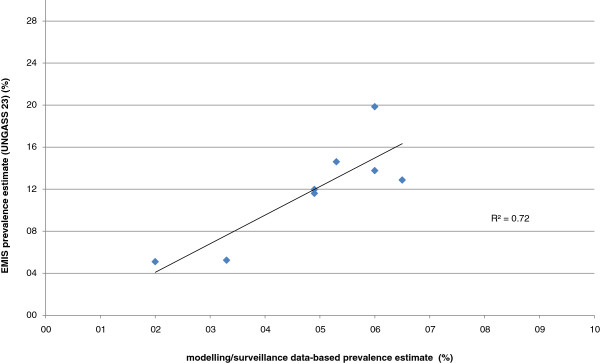
Correlation between modelling/ surveillance system data-based HIV prevalence estimates for MSM and self-reported HIV prevalence from EMIS.

From the remaining 29 countries, estimates for seven countries (Denmark, Germany, Greece, Luxemburg, Netherlands, Norway, and United Kingdom) were based on surveillance data and sophisticated (Germany, Netherlands, UK) or relatively simple modelling approaches. The simple approaches consisted of the number of diagnosed MSM in medical care plus an estimated number of undiagnosed infections (25%) divided by 3% of the adult male population (aged 15–64 years). The correlation between these modelled estimates and EMIS estimates for these seven countries was strong (R^2^=0.72; see Figure 2).

**Figure 3:**
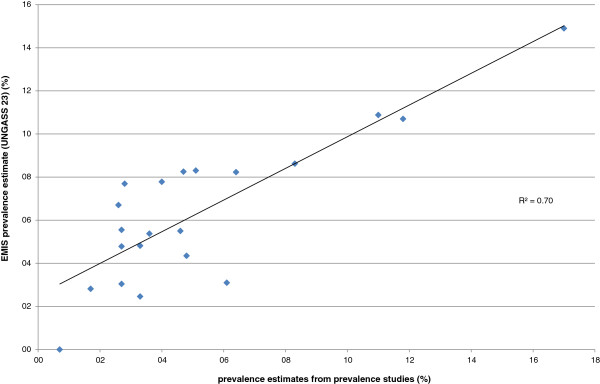
Correlation between results of specific HIV prevalence studies in MSM and self-reported HIV prevalence from EMIS.

One of the 22 countries with HIV prevalence studies had an estimate based on self-reported HIV status. For others, detailed information on methodology was not always available. For six countries data were based on a collaborative European study (SIALON I) using time location sampling in six cities (Barcelona, Bratislava, Bucharest, Ljubljana, Prague, and Verona [7]), the other estimates are based on respondent driven sampling, snowball sampling, or other less formalized venue based sampling methods. The correlation between the prevalence study estimates and the EMIS prevalence estimates was high (R^2^=0.70; see Figure 3).

**Figure 4:**
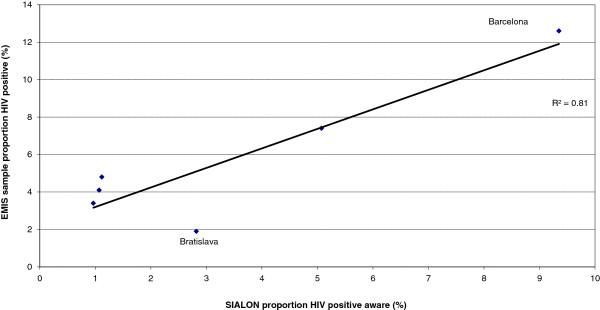
**Correlation between prevalence of diagnosed HIV in participants of the six-city.** Time Location Sampling study SIALON 1 and self-reported HIV prevalence from EMIS participants living in the respective cities.

Among the countries with larger discrepancies were countries with very small EMIS sample sizes (Macedonia, Bosnia) and low prevalence in direct prevalence studies. Discrepancies were also observed for some of the cities participating in the SIALON I study. However, if for these cities the EMIS prevalence estimates were correlated with the proportion of already diagnosed HIV infections in the study participants the correlation coefficient between the two measures further increased (R^2^ from 0.73 to 0.81; see Figure 4), with Bratislava as an outlier for both approaches.

Table 1 presents the surveillance system-derived HIV prevalence estimates among MSM for 29 countries together with a short description of the sources, the proportion of those ever tested who were living with diagnosed HIV, and the calculated SSD factor indicating the discrepancy between HIV prevalence measured in EMIS and pre-existing estimates.

More detailed EMIS data are presented in an Additional file 1: Table S1 and include: (1) total number of participants per country; (2) number ever tested for HIV; (3) number living with diagnosed HIV; (4) proportion of those never tested or not tested within the last 12 months who reported unprotected anal intercourse with any partner of unknown or discordant HIV status.

## Discussion

Self-reported prevalence rates of HIV diagnosis in a large Internet convenience sample of European MSM correlate strongly with prevalence estimates derived from direct prevalence studies using different sampling methods. This argues for at least partly similar self-selection biases in the different types of studies. Discrepancies between EMIS and pre-existing estimates can be due to:

•  different time points of measurement - most prevalence studies were conducted earlier than the EMIS survey (e.g. up to six years earlier in Poland);

•  measurement errors due to small sample sizes, particularly in smaller countries and countries with low estimated HIV prevalence among MSM (e.g. Bosnia-Herzegovina, Macedonia);

•  different self-selection biases in direct prevalence studies and self-reported prevalence in an Internet survey - this may apply particularly to countries with high degrees of HIV-related stigma as it is conceivable that in these countries MSM already diagnosed with HIV may be less inclined to participate in direct seroprevalence studies because of concerns regarding confidentiality, while they may be more inclined to participate in an anonymous Internet survey.

•  Of particular note, in four of five countries in which self-reported HIV prevalence in EMIS samples is lower than prevalence estimates from direct prevalence studies, these studies used Time Location Sampling in gay venues, suggesting that in those countries MSM visiting such venues might have a higher probability to be infected with HIV than participants of an internet survey.

The discrepancy between EMIS prevalence and the prevalence values derived from more sophisticated modelling approaches like Multiparameter Evidence Synthesis (MEPS) or back-calculation based on clinical staging and CD4 cell count at HIV diagnosis in this analysis was approximately 2 to 2.5-fold. This could mean that either the size of the total MSM population is overestimated in the respective models or that HIV positive participants are about two times as likely to participate in the EMIS survey in the respective countries. The Netherlands are an outlier in this regard with a calculated SSD of 3.3. Apart from the quite high EMIS prevalence estimate of 19.7%, also other sample characteristics like the highest median age of all samples argue for a possibly higher self-selection bias for Dutch EMIS participants. A possible explanation for a higher self-selection bias than other neighbouring countries could be the high frequency of Internet based behaviour surveys in the Netherlands (once yearly), possibly resulting in a kind of survey fatigue which may be more pronounced in younger, HIV negative or untested MSM.

A more thorough analysis and discussion of the issue of MSM population size and self-selection biases in internet surveys is beyond the scope of this paper. We would like to refer to another paper which compares self-reported newly diagnosed HIV infections in national EMIS samples and data on newly diagnosed HIV among MSM reported in the national surveillance systems, in which MSM population size estimations and how they relate to survey-surveillance discrepancies are addressed (Marcus U, et al.: Estimating the size of the MSM populations for 38 European countries by calculating the survey-surveillance discrepancies (SSD) between self-reported new HIV diagnoses from the European MSM Internet Survey (EMIS) and surveillance-reported HIV diagnoses among MSM in 2009. Submitted and under review).

There are several limitations to this analysis. First, the sample size of several national samples in EMIS was too small to make reliable HIV prevalence estimates. Secondly, there is no agreed definition of MSM and the size of the population is unknown for most countries. This means we cannot be confident that the relative prevalence rates from EMIS and specific prevalence studies refer to similar proportions of the adult male population. It is possible and conceivable that prevalence rates e.g. in UK, Germany, and the Netherlands refer to 3% of the adult male population, while prevalence rates in Poland, Romania, Bulgaria, Russia, and Ukraine refer to 1% or 1.5% of the adult male population.

## Conclusions

To summarize, EMIS, the first Pan-European MSM Internet survey, is a study that allows triangulation of data for almost all European countries. We can demonstrate a high correlation between self-reported HIV prevalence in EMIS and existing national prevalence studies using different sampling methods, however, self-reported prevalence in EMIS seems to be consistently higher than prevalence in other types of studies. Taking a likely systematic over-estimate into account, Pan-European community based open access Internet surveys can be cost-effective alternatives to national prevalence studies for generating comparable HIV prevalence estimates. Determination of the undiagnosed fraction of infections can better be achieved by studies collecting biological samples and behavioural data. However, variability of self-selection biases between studies in different countries and using different sampling methods may hamper cross-country comparisons of respective study results.

## Endnotes

^a^This measure may only be an approximation to the real prevalence because (a) the prevalence of undiagnosed HIV in those never tested may not be the same as in those who have ever tested, and (b) some men whose last HIV test was negative will have sero-converted since that test.

^b^For communication between ECDC and EU member states on surveillance data, disease-specific contact points are nominated. Usually these are the institutions responsible for national infectious disease surveillance. For verification of Russian data the Russian EMIS partners have been contacted.

## Competing interests

The authors declare that they have no competing interests.

## Authors’ contributions

All authors participated in the design of the survey tool. In addition: UM instigated the project, led the study design and drafted the manuscript. FH participated in the study design, constructed the online survey and contributed to the manuscript; PW participated in the study design, co-ordinated the survey promotion and contributed to the manuscript; AJS participated in the study design, coordinated the study and the EMIS Network and contributed to the manuscript. All authors approved the final manuscript.

## Authors’ information

The EMIS Network consists of Associated Researchers: Rigmor A. Berg (Norwegian Knowledge Centre for the Health Services, Oslo); Michele Breveglieri (Regione del Veneto, Verona); Laia Ferrer (CEEISCat, Barcelona); Percy Fernández-Davila (CEEISCat, Barcelona); Cinta Folch (CEEISCat, Barcelona); Martina Furegato (Regione del Veneto, Verona); Ford Hickson (Sigma Research, London); Harm J. Hospers (University College Maastricht); Ulrich Marcus (Robert Koch Institute, Berlin); David Reid (Sigma Research, London); Axel J. Schmidt (Robert Koch Institute, Berlin); Todd Sekuler (Robert Koch Institute, Berlin); Peter Weatherburn (Sigma Research, London)

National collaborating partners of the EMIS Network: Aids-Hilfe Wien (Austria); Facultés Universitaires Saint-Louis, Institute of Tropical Medicine, Ex Aequo, Sensoa, Arc-en-ciel (Belgium); Vstrecha (Belarus); National Centre of Infectious and Parasitic Diseases, Queer Bulgaria Foundation (Bulgaria); Charles University, Institute of Sexology (Czech Republic); Research Unit in Behaviour & Social Issues (Cyprus); University of Zagreb, Faculty of Humanities and Social Sciences (Croatia); Statens Serum Institut, Department of Epidemiology, stopaids (Denmark); National Institute for Health Development (Estonia); University of Tampere, Department of Nursing Science, Finnish AIDS council (Finland); Institut de veille sanitaire (InVS), AIDeS, Act UP Paris, Sida Info Service, Le kiosque, The Warning (France); Berlin Social Science Research Center (WZB), Deutsche AIDS-Hilfe (DAH), Federal Centre for Health Education, Cologne (BZgA) (Germany); Positive Voice (Greece); Hungarian Civil Liberties Union, Háttér (Hungary); Gay Men's Health Service, Health Services Executive (Ireland); University of Bologna, Italian Lesbian and Gay Association (Arcigay), Instituto Superiore di Sanità (National AIDS Unit)(Italy); The Infectiology Center of Latvia, Mozaika (Latvia); Center for Communicable Diseases and AIDS (Lithuania); GenderDoc-M (Moldova); schorer (Netherlands); Norwegian Knowledge Centre for the Health Services, The Norwegian Institute of Public Health (Norway); National AIDS Centre, Lamda Warszawa (Poland); GAT Portugal, University of Porto, Medical School, Inst. of Hygiene and Tropical Med. (Portugal); PSI Romania (Romania); PSI Russia (Russia); Safe Pulse of Youth (Serbia); OZ Odyseus (Slovakia); National Institute of Public Health, SKUC-Magnus, Legebitra, DIH (Slovenia); National Centre of Epidemiology, stopsida, Ministerio de Sanidad, Política Social e Igualdad (Spain); Malmö University, Health and Society, RFSL, National Board of Health and Welfare (Sweden); Institut universitaire de médecine sociale et preventive, Aids-Hilfe Schweiz (Switzerland); Turkish Public Health Association, Siyah Pembe Üçgen İzmir, KAOS-GL, Istanbul-LGBTT (Turkey); Gay Alliance, Nash Mir, LiGA, Nikolaev (Ukraine); City University London, Department for Public Health, Terrence Higgins Trust and the CHAPS partners including GMFA, The Eddystone Trust, Healthy Gay Life, The Lesbian and Gay Foundation, The Metro Centre London, NAM, Trade Sexual Health, Yorkshire, MESMAC (United Kingdom).

European Collaborating Partners: International Gay and Lesbian Organization (ILGA); European AIDS Treatment Group (EATG); PlanetRomeo.com; Manhunt and Manhunt Cares

## Pre-publication history

The pre-publication history for this paper can be accessed here:

http://www.biomedcentral.com/1471-2458/12/978/prepub

## Supplementary Material

Additional file 1 **Table S1. **Country data on EMIS sample size, number ever tested for HIV, self-reported HIV prevalence, and proportion with reported UAI risk in the previous 12 months among those who have never tested for HIV. Sorting by increasing probability of underestimating prevalence in the EMIS sample by self-reported prevalence (based on proportion ever testing among respondents with UAI-risk in previous 12 months). (PDF 10 kb) Click here for file
