# Supplementary material for: Prevalence of HIV among MSM in Europe: comparison of self-reported diagnoses from a large scale internet survey and existing national estimates
Source: BMC Public Health. 2012 Nov 14;12:978. doi: 10.1186/1471-2458-12-978 (PMC3526585; doi:10.1186/1471-2458-12-978)
Supplement: Additional file 1 — Table S1. Country data on EMIS sample size, number ever tested for HIV, self-reported HIV prevalence, and proportion with reported UAI risk in the previous 12 months among those who have never tested for HIV. Sorting by increasing probability of underestimating prevalence in the EMIS sample by self-reported prevalence (based on proportion ever testing among respondents with UAI-risk in previous 12 months). (PDF 10 kb) [file 1471-2458-12-978-S1.pdf]

Additional Table: Country data on EMIS sample size, number ever tested for HIV, self-reported HIV prevalence, and proportion with reported UAI risk in the previous 12 months among those who have never tested for HIV. Sorting by increasing probability of underestimating prevalence in the EMIS sample by self-reported prevalence (based on proportion ever testing among respondents with UAI-risk in previous 12 months)

		Sample size (N)	Ever tested for HIV	proportion ever tested	Diagnosed HIV+ (N)	Diagnosed HIV+ among tested (%)	UAI reported in previous 12 months among respondents not tested in previous 12 months*	UAI reported in previous 12 months among respondents never tested**
fr	France	11,197	9,299	83.0%	1,180	12.7%	54.5%	28.7%
ch	Switzerland	5,045	3,979	78.9%	456	11.5%	60.9%	30.5%
be	Belgium	4,000	3,210	80.3%	338	10.5%	57.5%	31.8%
dk	Denmark	1,749	1,286	73.5%	154	12.0%	63.4%	32.3%
nl	Netherlands	3,810	2,997	78.7%	595	19.9%	63.1%	34.6%
se	Sweden	3,149	2,343	74.4%	151	6.4%	66.5%	35.1%
lu	Luxemburg	279	218	78.1%	30	13.8%	57.8%	37.8%
es	Spain	13,136	9,612	73.2%	1,170	12.2%	61.0%	38.2%
uk	United Kingdom	17,739	12,647	71.3%	1,847	14.6%	64.6%	38.9%
at	Austria	4,112	3,054	74.3%	221	7.2%	69.4%	43.5%
it	Italy	15,915	11,275	70.8%	1,096	9.7%	66.8%	44.7%
ru	Russia	5,055	3,723	73.6%	321	8.6%	67.0%	44.7%
pt	Portugal	5,193	3,723	71.7%	405	10.9%	64.8%	44.8%
bg	Bulgaria	1,045	650	62.2%	16	2.5%	63.9%	46.1%
no	Norway	2,100	1,373	65.4%	72	5.2%	71.1%	46.2%
cy	Cyprus	267	156	58.4%	3	1.9%	74.7%	47.0%
de	Germany	54,774	37,764	68.9%	4,379	11.6%	73.0%	48.0%
ie	Ireland	2,196	1,379	62.8%	131	9.5%	72.5%	48.2%
mt	Malta	116	80	69.0%	2	2.5%	71.4%	48.6%
ee	Estonia	590	355	60.2%	10	2.8%	75.6%	52.4%
gr	Greece	2,964	1,857	62.7%	239	12.9%	77.0%	53.5%
fi	Finland	2,028	1,265	62.4%	65	5.1%	78.8%	54.1%
by	Belarus	368	230	62.5%	7	3.0%	78.0%	55.9%
tk	Turkey	1,813	895	49.4%	27	3.0%	76.0%	57.6%
hu	Hungary	2,069	1,189	57.5%	66	5.6%	74.1%	57.7%
pl	Poland	2,776	1,709	61.6%	141	8.3%	78.4%	58.3%
mk	Macedonia	118	65	55.1%	5	7.7%	72.4%	58.6%
ua	Ukraine	1,719	1,009	58.7%	83	8.2%	76.2%	61.4%
si	Slovenia	982	507	51.6%	26	5.1%	80.2%	61.5%
md	Moldowa	117	69	59.0%	3	4.3%	81.1%	62.2%
lv	Latvia	701	347	49.5%	27	7.8%	81.1%	63.5%
ro	Romania	2,338	1,168	50.0%	60	5.1%	78.5%	64.3%
rs	Serbia	1,108	577	52.1%	31	5.4%	80.9%	65.6%
cz	Czech	2,437	1,339	54.9%	66	4.9%	82.8%	68.1%
hr	Croatia	523	270	51.6%	13	4.8%	86.3%	68.1%
lt	Lithuania	591	251	42.5%	12	4.8%	87.8%	69.3%
sk	Slovakia	590	274	46.4%	6	2.2%	84.5%	70.6%
ba	Bosnia	150	65	43.3%	0	0.0%	85.7%	73.8%
**Min**				**42.5%**		**0.0%**	**54.5%**	**28.7%**
**Median**				**63.1%**		**7.5%**	**72.6%**	**51.2%**
**Max**				**83.0%**		**19.9%**	**87.8%**	**73.8%**

*proportion of EMIS respondents not yet diagnosed with HIV who reported UAI with any partner of unknown or discordant HIV status in the previous 12 months who have not been tested for HIV in the previous 12 months

**proportion of EMIS respondents not yet diagnosed with HIV who reported UAI with any partner of unknown or discordant HIV status in the previous 12 months who have never been tested for HIV
